# Comprehensive analysis of aerobic glycolysis-related genes for prognosis, immune features and drug treatment strategy in prostate cancer

**DOI:** 10.3389/fonc.2022.905888

**Published:** 2022-09-29

**Authors:** Wei He, Xiang He, Enhui Li

**Affiliations:** Urology and Nephrology Center, Department of Urology, Zhejiang Provincial People’s Hospital, Affiliated People’s Hospital, Hangzhou Medical College, Hangzhou, China

**Keywords:** prostate cancer, aerobic glycolysis, immune microenvironment, chemotherapy response, prognosis model

## Abstract

**Background:**

The dysregulated expression of aerobic glycolysis-related genes is closely related to prostate cancer progression and metastasis. However, reliable prognostic signatures based on aerobic glycolysis have not been well established.

**Methods:**

We screened aerobic glycolysis-related gene modules by weighted gene co-expression network analysis (WGCNA) and established the aerobic glycolysis-related prognostic risk score (AGRS) by univariate Cox and lasso-Cox. In addition, enriched pathways, genomic mutation, and tumor-infiltrating immune cells were analyzed in AGRS subgroups and compared to each other. We also assessed chemotherapeutic drug sensitivity and immunotherapy response among two subgroups.

**Results:**

An aerobic glycolysis-related 14-gene prognostic model has been established. This model has good predictive prognostic performance both in the training dataset and in two independent validation datasets. Higher AGRS group patients had better immunotherapy response. Different AGRS patients were also associated with sensitivity of multiple prostate cancer chemotherapeutic drugs. We also predicted eight aerobic glycolysis-related small-molecule drugs by differentially expressed genes.

**Conclusion:**

In summary, the aerobic glycolysis-derived signatures are promising biomarkers to predict clinical outcomes and therapeutic responses in prostate cancer.

## Introduction

Prostate cancer (PC) is the third most common cancer of all tumors and the second most common cancer in men worldwide. In 2020, globally, 1,414,259 people worldwide had PC and 375,304 patients died of PC (1). Although time-to-biochemical recurrence (BCR), Gleason score, and prostate-specific antigen (PSA) doubling time are important prognostic factors (2), these factors also have their limitations. Therefore, it is urgent to find a better method to predict tumor prognosis and therapy response.

One of the main reasons why tumors are difficult to treat is that tumor cells have a robust ability to survive in harsh environments by changing their energy metabolism, which is known as “metabolic reprogramming”. Metabolic reprogramming is generally considered a downstream consequence of tumor development. However, increasing evidence suggests that metabolism in turn can support oncogenic signaling to promote tumor malignancy (3–5). Aerobic glycolysis (AG) is a typical example, which is characterized by increase in glucose uptake and the production of lactate and was initially described as the “Warburg effect” (6). It can provide the energy and material needs of the rapid growth of tumor cells (7). Many previous studies have shown that aerobic glycolysis contributes to many malignant features of tumors (8–10).

**Figure 1:**
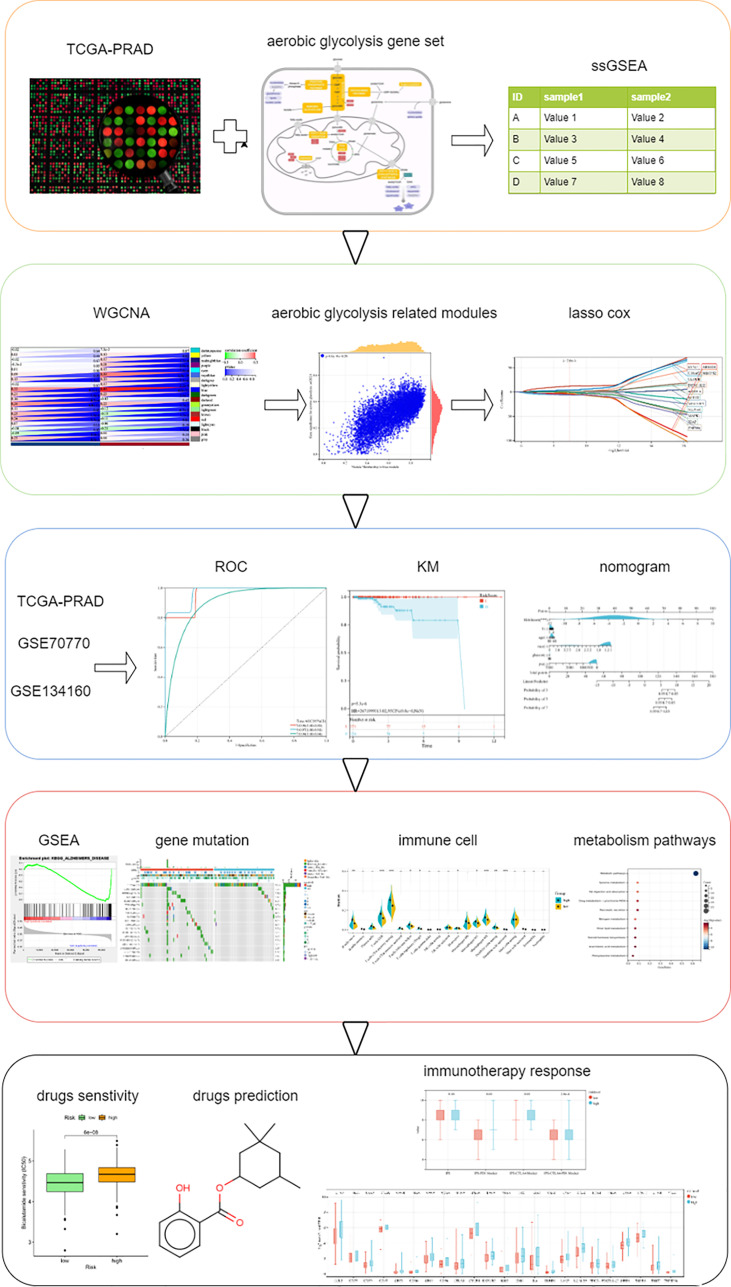
Schematic diagram of the study design.

In our study, we performed AG-level estimation on samples in The Cancer Genome Atlas (TCGA, https://portal.gdc.cancer.gov/), screened out AG-related genes using weighted gene co-expression network analysis (WGCNA), and finally established a 14-gene signature and validated it in multiple independent datasets. In addition, we also analyzed the response to immunotherapy by IPS scoring and immune checkpoint molecule expression analysis. Multiple chemotherapeutic drugs were related with AG. We predicted some small-molecule drugs showing therapeutic effects on PC. The analysis procedures in this study are summarized in **Figure 1**.

## Materials and methods

### Data acquisition

Public gene-expression data and clinical information were searched in the Gene Expression Omnibus (GEO; https://www.ncbi.nlm.nih.gov/gds/) and TCGA databases. The eligible criteria of the dataset included 1) owning over survival (OS) time information and 2) at least 100 PC samples. We removed the datasets that did not meet the criteria by checking them one by one carefully and gathered two patient cohorts from GEO. In total, we gathered three patient cohorts for this study: TCGA-PRAD, GSE134160 (11), and GSE70770 (12). Two GEO datasets were processed expression matrices downloaded from GEO. All microarray data included in our study were log2 transformed and Z-score transformed. Data files of count expression of TCGA-PRAD and clinical data were downloaded by using the “TCGAbiolinks” package in R (13). The RNA-seq data were converted to transcripts per million (TPM). We used TCGA-PRAD dataset as the training dataset because of its large sample size and rich genomic information. Moreover, another two datasets from different platforms were used as independent validation sets, namely, GSE134160 and GSE70770. TCGA-PRAD somatic mutation data were downloaded from TCGA using the package “TCGAbiolinks” in R. Somatic mutation data were analyzed using R package “maftools” (14). The multiple gene sets were obtained from the MSigDB database (http://www.gsea-msigdb.org/gsea/msigdb/index.jsp). A total of 2,071 metabolism-related genes were obtained from the ccmGDB database (15). We downloaded the IPS of TCGA-PRAD cohort from The Cancer Immunome Atlas (TCIA) (https://tcia.at/home).

### Screening of aerobic glycolysis-related genes and establishment of risk model

The pathway level of multiple gene sets in TCGA-PRAD were quantified based on a single-sample gene set enrichment analysis (ssGSEA) algorithm by the “GSVA” package in R (16); the method parameter in the algorithm was set to GSVA and ssgsea. From this, we got the scores of multiple gene sets in all samples. Based on the results above, WGCNA was performed using the “WGCNA” R package (17). First, we used TPM gene expression profiles and calculated the median absolute deviation of each gene separately, obtained the top 25% genes for further analysis, and removed outlier genes and samples using the goodSamplesGenes method of WGCNA and further used WGCNA to build a scale-free co-expression network. We calculated that the highest β value is 9, and the minimum number of genes in the module was set to 50. The gene network of the key module was extracted, and the network map was drawn using Cytoscape 3.6.1, and then the degree data of each RNA in the network were downloaded. Then, univariate Cox regression analysis was performed on the screened gene expression profiles after the Z-score. The genes with P < 0.01 (Wald test) were defined to be related to OS. Then, we used the R software package “glmnet” to perform the LASSO-Cox analysis. In addition, we also set up 10-fold cross-validation to obtain the optimal model. The AG risk score (AGRS) of each sample was obtained by multiplying the obtained coefficient by the gene expression value.

### Aerobic glycolysis model validation

The optimal cutoff value was confirmed by R package “maxstat”, setting the minimum number of grouping samples to be greater than 25% and the maximum number of samples to be grouped less than 75%. The patients were divided into high-risk group and low-risk group, and the Kaplan–Meier (KM) method with the log-rank test was used to further analyze the prognostic differences between the two groups. The prognostic or predictive accuracy of gene panels was assessed using time-dependent receiver operating characteristic (ROC) analysis. The area under the curve (AUC) at different cutoff times was used to measure the accuracy of prognosis or prediction. We integrated prognostic and clinicopathological features to construct a nomogram to visually assess the patients’ 3-, 5-, and 7-year survival rate in TCGA-PRAD.

### Estimation of immune cells

The proportions of 22 immune cell types in PC samples were estimated using the CIBERSORT algorithm (https://cibersortx.stanford.edu/) with batch-corrected mode, relative mode, and 1,000 permutations of b mode (18). The Wilcoxon test was used to find the significantly different immune cells among different groups.

### Gene enrichment analysis and gene set enrichment analysis

For gene set functional enrichment analysis, we used the KEGG rest API (https://www.kegg.jp/kegg/rest/keggapi.html) to obtain the latest gene annotations of the Kyoto Encyclopedia of Genes and Genomes (KEGG) Pathway and performed enrichment analysis sing the R package “clusterProfiler” to obtain the results of gene set enrichment. A P value of < 0.05 was considered statistically significant. We downloaded the GSEA software (version 4.3) from the gene set enrichment analysis (GSEA: http://software.broadinstitute.org/gsea/index.jsp) website and set the “c2.cp.kegg.v7.1.symbols.gmt gene sets” as the reference set. All samples were divided into two groups based on the AGRS median, which is used as input for the phenotype file. A NOM P-value < 0.05 was considered statistically significant.

### Additional bioinformatic and statistical analyses

The DESeq2 package in R was used to identify the differentially expressed genes (19). Differences between the two groups were compared using violin plots, and the Wilcoxon test was used. The half-maximal inhibitory concentration (IC50) was estimated by R package “pRRophetic” (20). The Connectivity Map (CMap, https://clue.io/) was used to predict the small candidate molecules based on differentially expressed genes. All of the above analyses were performed using the R software (version 4.0.2, http://www.rproject.org). Statistical significance was set at P < 0.05.

### Gene expression verification

To visualize the differences in clinical expression of key gene proteins, we investigated the expression of these genes in PC tissue and normal tissue in the Human Protein Atlas (HPA) database. Furthermore, to verify the expression levels of 14 mRNAs, we performed differential gene expression analysis on tumor tissues and normal tissues in TCGA-PRAD. In addition, we collected 20 pairs of PC samples and adjacent normal samples in our hospital to analyze the RNA expression differences of these genes using rt-PCR, all of which were approved by the patients’ informed consent and the ethics committee of Zhejiang Provincial People’s Hospital. TRIzol (Thermo Fisher, USA) was used to extract the total RNA in the sample, and the “Reverse Transcription RR047A Kit (Takara, Japan) was used to convert it into cDNA. Finally, the RR820A kit (Takara, Japan) was used to perform rt-PCR analysis on the 7900HT system (Thermo Fisher, USA), and the ACTB gene was used as the internal reference gene to calculate the expression of hub genes with each pair of tissues.

## Result

### Characteristics of different aerobic glycolysis levels

**Figure 2:**
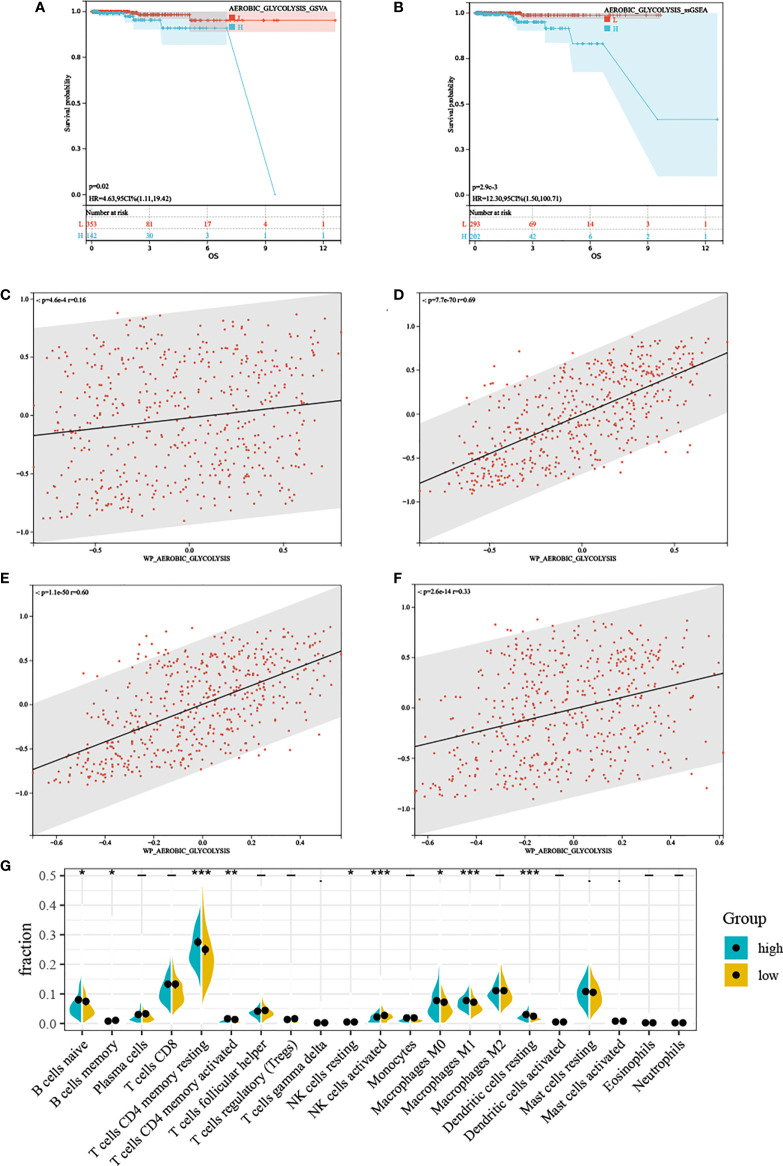
The characteristics between different aerobic glycolysis levels. **(A, B)** Kaplan–Meier plots indicating high aerobic glycolysis level patients have a shorter survival time. **(C)** The scatter plot shows a significant correlation between the glutamate metabolism and the aerobic glycolysis metabolism. **(D)** The scatter plot shows a significant correlation between the pentose phosphate metabolism and the aerobic glycolysis metabolism. **(E)** The scatter plot shows a significant correlation between the lactic acid metabolism and the aerobic glycolysis metabolism. **(F)** The scatter plot shows a significant correlation between the fatty acid metabolism and the aerobic glycolysis metabolism. **(G)** Relative proportion of infiltrating immune cells in low- and high- aerobic glycolysis level prostate cancer patient of TCGA-PRAD cohort. “*, **, ***” stands for statistically significant.

Regardless of the method used to assess AG, high AG was associated with poorer prognosis (**Figures 2A, B**), which was inconsistent with our previous thought. These findings showed that AG was a risk factor for overall survival in PC. Based on the ssGSEA results, we divided TCGA-PRAD patients into high and low groups by median. GSEA results showed that the high AG level group was mainly enriched in tumor-related pathways, immune-related pathways, and various metabolic-related pathways (**Supplementary Materials**). The associated metabolic pathways include alanine aspartate and glutamate metabolism, pentose phosphate pathway, glycosphingolipid biosynthesis-lacto and neolacto series, and biosynthesis of unsaturated fatty acids, and the correlation analysis also proved that aerobic glycolysis is related to these three energy metabolisms (**Figures 2C–F**). Immune-related pathways mainly include leukocyte trans-endothelial migration, T-cell receptor signaling pathway, natural killer cell-mediated cytotoxicity, and B-cell receptor signaling pathway. We performed CIBERSORT, and the violin plot showed significant differences in a variety of immune cells including B cells, T cells CD4, T cell gamma delta, NK cells, macrophages M0, macrophages M1, dendritic cells resting, and mast cells (**Figure 2G**).

**Figure 3:**
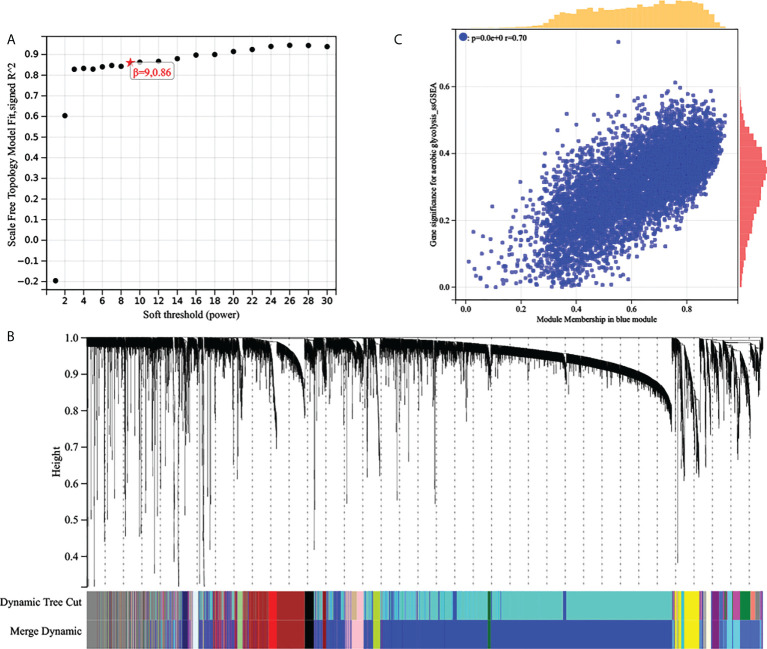
**(A)** Analysis of the scale‐free fit index of the matrix. **(B)** Clustering dendrograms of top 25% genes, with dissimilarity based on topological overlap, together with assigned module colors. **(C)** Scatter plots of the gene significance and module membership in blue modules. The x-axis indicates the module membership (MM) which quantifies how close a gene is to a given module. The y-axis indicates the gene significance which is correlated with clinical traits.

**WGCNA:** The top 25% most variable genes were used for WGCNA, and 18 modules were identified (**Figures 3A, B**). Among these modules, the blue modules (r = 0.49, P = 4e-31) exhibited the highest correlation with AG (**Figure 3C**). Module membership (MM) was set to 0.8, and gene significance (GS) was set to 0.25. According to this criterion, we screened out the hub genes in which the blue modules were related to the AG trait. The gene network of the blue module was extracted and the network was built using Cytoscape, and the genes with a degree greater than 20 were extracted. We took the intersection of the two obtained gene sets and finally obtained 513 genes.

### Establishment and validation of the aerobic glycolysis prognostic signature for overall survival in prostate cancer

**Figure 4:**
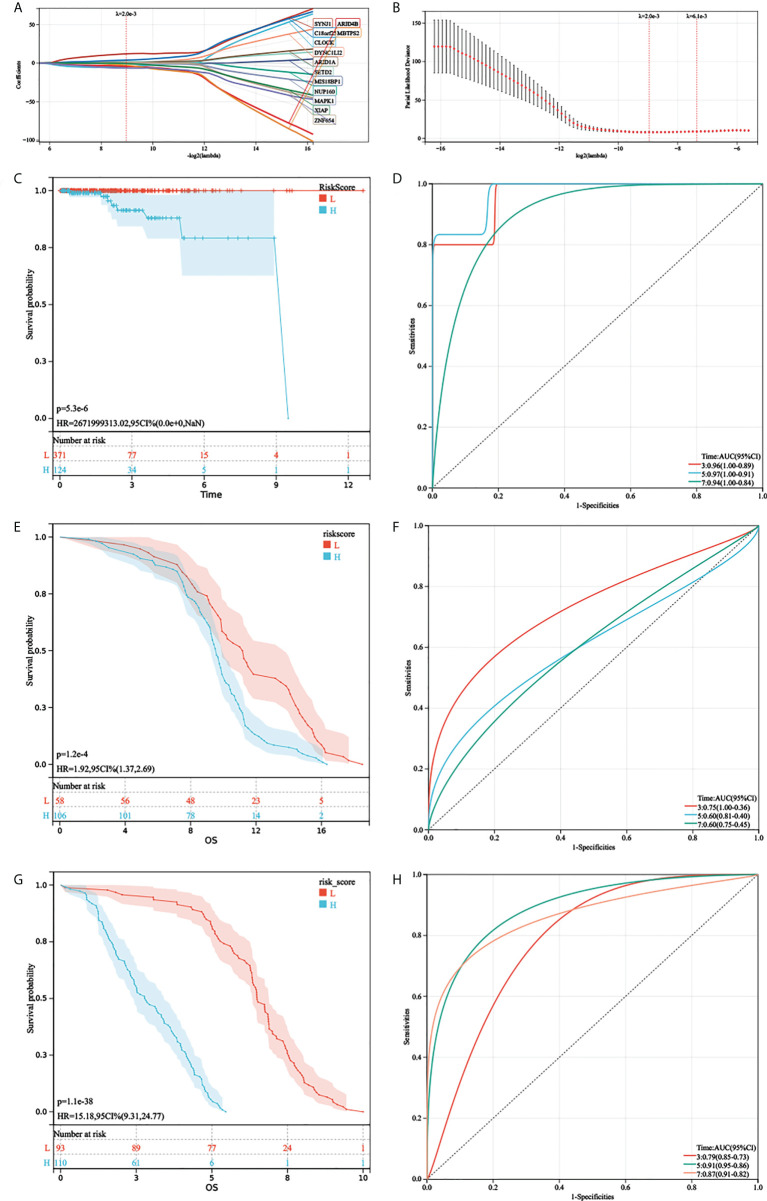
Development and validation of AGRS in TCGA-PRAD cohort. **(A, B)** The LASSO Cox regression algorithm was used to identify the most robust prognostic genes. **(C, D)** Patients were divided into high-risk and low-risk subgroups based on best cutoff. Kaplan–Meier analysis demonstrated that patients with higher AGRS exhibited worse overall survival in TCGA-PRAD, ROC curves showing the predictive efficiency of the AGRS on the 3-, 5-, and 7-year survival rate. **(E, F)** Kaplan–Meier analysis and ROC curves in the GSE135160 cohort. **(G, H)** Kaplan–Meier analysis and ROC curves in the GSE70770 cohort.

First, gene expression data were transformed by Z-score. Then, the univariate Cox regression method was used to identify genes that were associated with prognosis and we found 24 genes with P < 0.01. Subsequently, the LASSO Cox algorithm was applied to identify the most robust prognostic genes. The optimal λ value of 0.00200006822207054 was selected (**Figures 4A, B**). Finally, the risk scores of each PC patient were calculated using the following method: AGRS = -4.47765657690985*CLOCK+1.29384446467155*NUP160+0.846130542021257*MIS18BP1+1.06447461483067*DYNC1LI2-0.137000461457667*MAPK1+1.57474598539145*SETD2-4.05360227520656*ARID4B+0.566257977207755*ARID1A+1.13167548982397*ZNF654+12.0526647311552*SYNJ1+3.88187142257071*C18orf25-2.81564312424355*MBTPS2-0.577996066831003*XIAP-6.43690052943485*ZNF678. The K-M plot demonstrated that the high AGRS group had unfavorable OS compared with the low AGRS group (P =5.3e-6, **Figure 4C**). Moreover, the AUC values for 3-, 5-, and 7-year OS were 0.96, 0.97, and 0.94 (**Figure 4D**), respectively, which were good classification results. To verify the effectiveness of this model, a risk score was calculated in the GSE134160 cohort and GSE70770 cohort. Surprisingly, results from two independent cohorts revealed that the high-risk group had a worse prognosis than the low-risk group (**Figures 4E, G**). Moreover, the AUC of AGRS in the two independent cohorts also proved that the risk score model has an excellent prognostic prediction effect (**Figures 4F, H**).

### Aerobic glycolysis prognostic signature can be utilized as an independent prognostic factor in prostate cancer

Considering the importance of aerobic glycolysis in the prognosis of prostate cancer, we further analyzed the relationship between 14 aerobic glycolysis prognostic signatures and clinical features of prostate cancer including age, pathological T stage, race, and Gleason score. Among them, only the ARGS differed significantly between different Gleason scores (**Figures S1–S4**).

**Figure 5:**
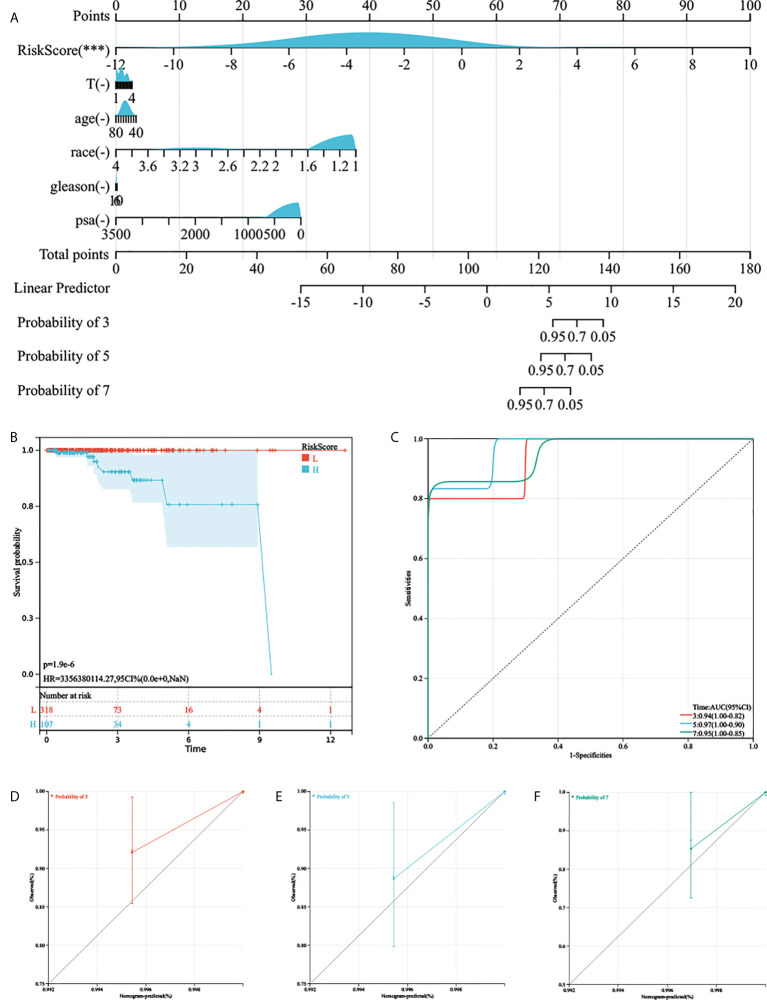
The nomogram was generated to improve risk stratification and estimate survival probability. **(A)** The comprehensive nomogram for predicting probabilities of PC patients with 3-, 5-, and 7-year OS in TCGA-PRAD cohort. **(B, C)** Kaplan–Meier analysis and ROC curves of 3-, 5-, and 7-year OS for this nomogram. **(D–F)** The calibration plots for predicting PC patients with 3-, 5-, and 7-year OS in TCGA-PRAD cohort.

As the AGRS was significantly correlated with high malignancy, we sought to determine whether the AGRS was a clinically independent prognostic factor for PC patients through multivariate Cox regression analyses. The AGRS and other clinical features, including age, Gleason, pathological T stage, and prostate-specific antigen (PSA), were enrolled as covariates to perform the analysis. By combining the above prognostic factors, we constructed a nomogram that serves as a clinically relevant quantitative method by which clinicians can predict mortality in PC patients (**Figures 5A, B**). Each patient will be assigned a total of points by adding points for each prognostic parameter. The overall C-index of the model is 0.96, 95% CI (0.90-1), P-value = 2.40e-44. The capacity of the nomogram to distinguish survival was tested using AUC values (**Figure 5C**). In the calibration analysis, the prediction lines of the nomogram for 3-, 5-, and 7-year survival probability were extremely close to the ideal performance (45° line) (**Figures 5D–F**), indicating a high accuracy of the nomogram.

### Comprehensive analyses of multiple characteristics between different risk groups

To illustrate the underlying biological characteristics of the different aerobic glycolysis risk groups, we performed GSEA in TCGA-PRAD cohort. The results showed that the high-risk group is mainly enriched in multiple immune-related pathways, including viral protein interaction with cytokine and cytokine receptor, natural killer cell-mediated cytotoxicity, IL-17 signaling pathway, Th1- and Th2-cell differentiation, Th17-cell differentiation, T-cell receptor signaling pathway, leukocyte trans-endothelial migration, and PD-L1 expression and PD-1 checkpoint pathway in cancer (**Supplementary Materials**). In addition, the low-risk group was also significantly enriched in oxidative phosphorylation, ribosome and protein export, and thermogenesis (**Supplementary Materials**).

**Figure 6:**
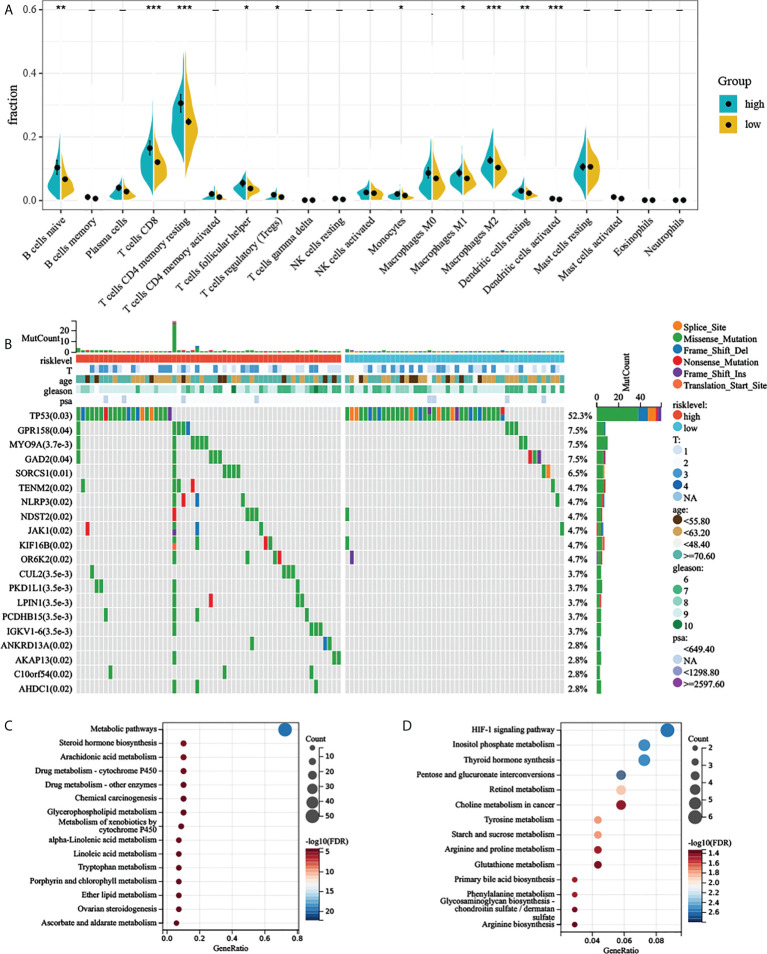
Comprehensive analyses of different risk groups. **(A)** Relative proportion of infiltrating immune cells in low- and high-AGRS prostate cancer patient of TCGA-PRAD cohort. **(B)** Top 20 most frequently mutated genes were illustrated in TCGA-PRAD. **(C, D)** Enrichment analysis of metabolic genes in different risk subgroups. “* ,**, ***” stands for statistically significant.

CIBERSORT along with the LM22 matrix was used to assess immune cell infiltration in the low- and high-risk groups of TCGA-PRAD. **Figure 6A** indicates that B cells, CD4+ and CD8+ T cells, macrophages, and mast cells were the predominant infiltrating immune cells in PC. We found that B cell naïve, T cells CD8, T cells CD4 memory resting, T cells CD4 memory activated, T cells follicular helper, T cells regulatory (Tregs), monocytes, macrophages M0, macrophages M1, macrophages M2, dendritic cells resting, and dendritic cells activated differently between the two groups (**Figure 6A**).

To investigate whether there was evidence of differences at the genomic level between low- and high-risk PC patients, we investigated the distribution differences of somatic alterations. Waterfall plots integrated with 20 highly variant mutant genes were utilized to show the mutation landscape. As shown in **Figure 6B**, among the top 20 mutated genes, the high-risk group appears to have a higher mutation rate relative to the low-risk group. The mutation rates of these top 20 mutated genes differed in both groups (**Figure 6B**). Although there were only 124 samples in the high-risk group and 371 samples in the low-risk group, the apparently high-risk group had a much higher mutation rate.

Previous studies have suggested that aerobic glycolysis significantly affects cellular metabolism (21). We therefore explored the metabolomic variations between the two risk groups by analyzing the expression of 2,071 metabolism-related genes (**Supplementary Materials**) obtained from the ccmGDB database (15). We first performed differential expression analysis between the two groups, log2 fold change was set to 0.5, and P-value was set to 0.05. By taking the intersection of differentially expressed genes and metabolism-related genes, we obtained 79 genes that were highly expressed in the high-risk group. Next, we carried out KEGG enrichment analysis of upregulated metabolism-related genes. The high-risk group showed enrichment of steroid hormone biosynthesis, arachidonic acid metabolism, alpha-linolenic acid metabolism, and many metabolism-related pathways (**Figures 6C, D** and **Supplementary Materials**). High-risk groups were also associated with multiple drug metabolism-related pathways (**Figure 6C**), which means AGRS may predict drug efficacy. We also found that the HIF-1 signaling pathway was also significantly enriched in the high-risk group (**Figure 6D**), which also demonstrated a close relationship between aerobic glycolysis and tumor hypoxia.

### ARGS-based treatment strategy for prostate cancer

**Figure 7:**
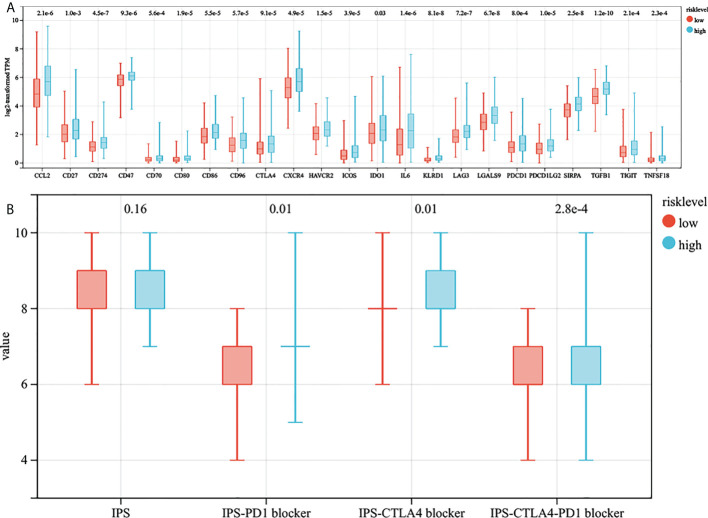
The estimation of two prognostic subtypes in immunotherapy response. **(A)** The expression of 23 immune checkpoint molecules in two prognostic subtypes (the y-axis indicates expression log2 transformed TPM). **(B)** The association between IPS and risk score.

We collected 23 immune checkpoint molecules and analyzed their gene expression differences between the two groups. The results showed that all immune checkpoint molecules were significantly overexpressed in the high-risk group (**Figure 7A**). In addition, the immunogenicity of two risk subgroups was evaluated by IPS analysis. Higher IPS scores were positively correlated with the increased immunogenicity (22). The IPS-CTLA4, IPS-PD1, and IPS-PD1-CTLA4 scores were higher in the high-risk group (**Figure 7B**). The above analysis results show that the high-risk group had a better response to immunotherapy.

**Figure 8:**
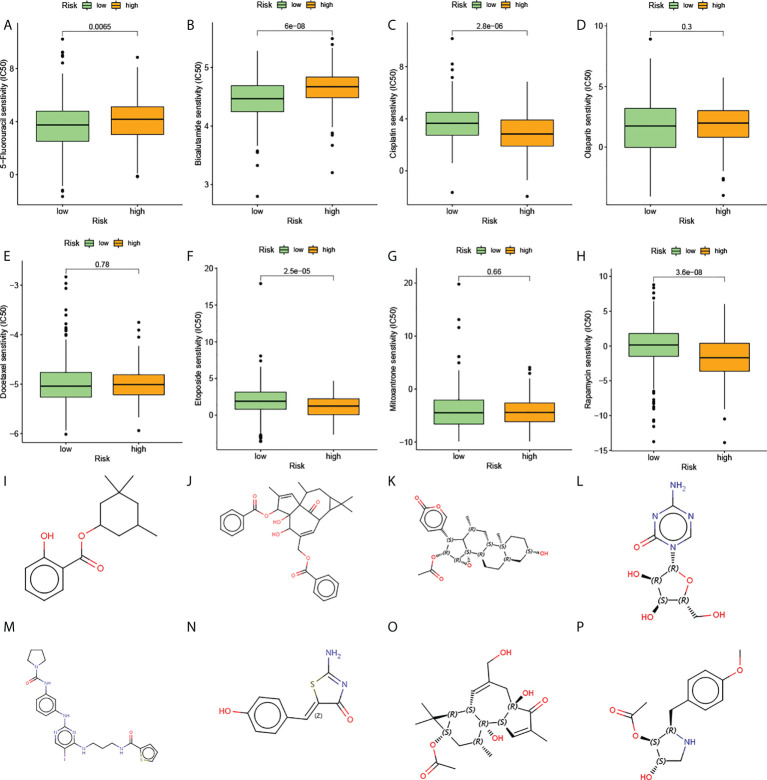
The estimation of chemotherapy response and potential therapeutic drugs for prostate cancer. **(A–H)** The chemotherapy response of two prognostic subtypes for eight common chemotherapy drugs. **(I–P)** The molecular structure of the eight small-molecule drugs for prostate cancer: **(I)** homosalate, **(J)** ingenol, **(K)** cinobufagin, **(L)** azacitidine, **(M)** BX-795, **(N)** mirin, **(O)** prostratin, and **(P)** anisomycin.

The above analysis of metabolic genes indicated that the high-risk group was associated with multiple drug metabolism pathways, which led us to consider whether AGRS could be used as a marker for predicting drug response. The Cancer Genome Project (CGP) database was used to predict the chemotherapeutic response of two subtypes to commonly used chemotherapeutic drugs. There are eight prostate cancer chemotherapy drugs in this database, of which five chemotherapy drugs are significantly different in the estimated IC50 between the two subgroups (**Figures 8A–H**). The high-risk patients were more sensitive to the anticancer drugs etoposide, cisplatin, and rapamycin. The low-risk patients were more sensitive to the anticancer drugs bicalutamide and 5-fluorouracil.

The CMap database was used to screen out small-molecule drugs showing therapeutic effects on PC. Based on the 169 upregulated and 88 downregulated genes, eight potential small-molecule drugs targeting genes were identified (**Figures 8I–P**). The structural diagrams of these molecules were also downloaded from CMap.

### Verification of 14 genes in this model

**Figure 9:**
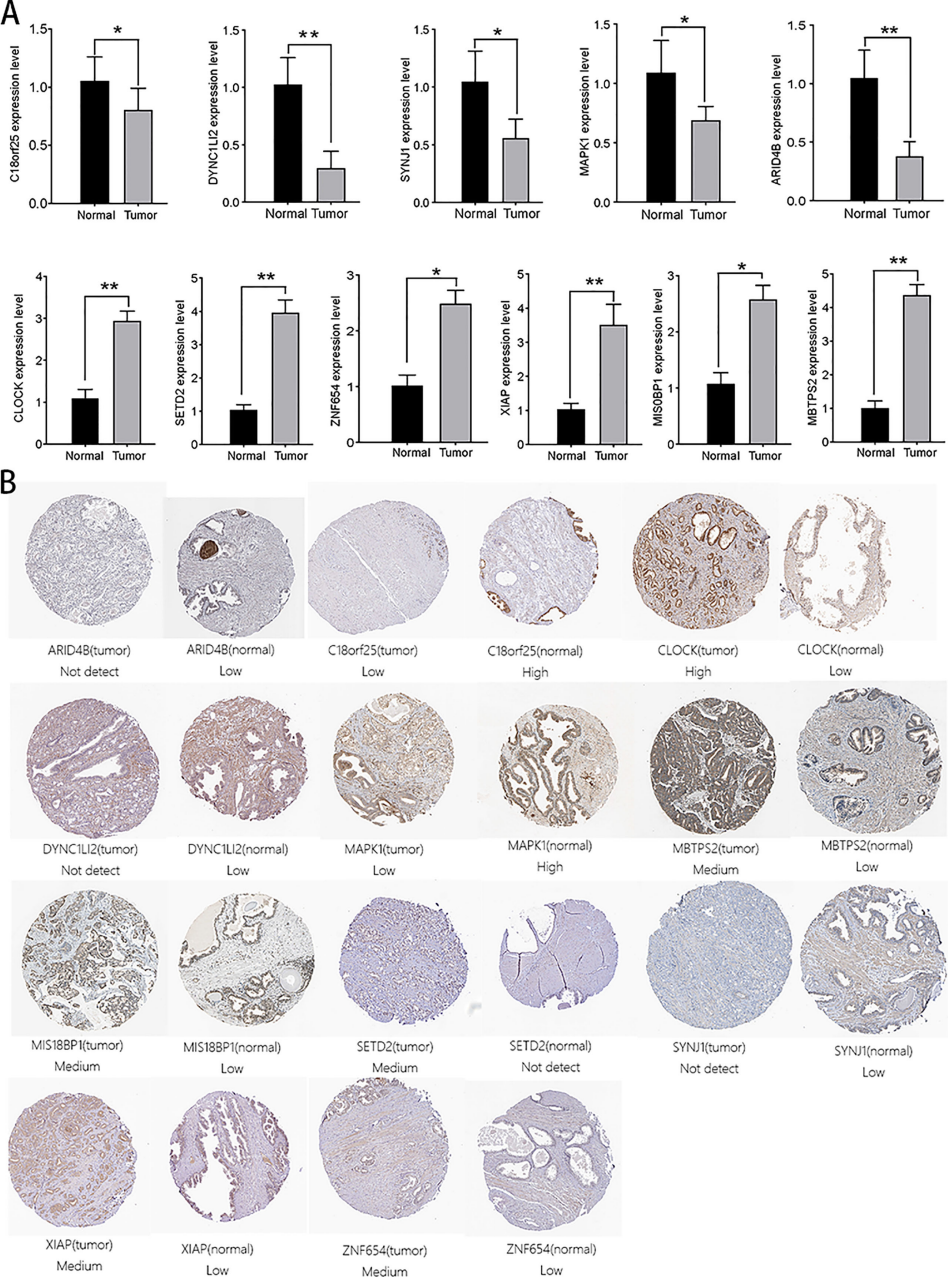
Verification of the expression of aerobic glycolysis-related signatures in normal and tumor tissues. **(A)** Immunohistochemistry of aerobic glycolysis-related signatures in normal prostate tissue and prostate cancer tissue. **(B)** rt-PCR verified the expression of aerobic glycolysis-related signatures in 20 pairs of prostate cancer clinical samples. All data are displayed as means ± SD; mean values for the normal group were normalized to 1.0; two-sided unpaired Student’s test was applied. **P < 0.01 and *P < 0.05 *vs*. normal group.

We verified the expression levels of 14 genes from protein and mRNA levels. In the 20 pairs of clinical samples we collected, rt-PCR results showed that ZNF678, ARID1A, XIAP, MIS18BP1, and MBTPS2 were highly expressed in tumor tissues. Moreover, C18orf25, NUP160, DYNC1LI2, SYNJ1, MAPK1, and CLOCK were lowly expressed in tumor tissues (**Figure 9A**). The HPA database suggested that C18orf25, DYNC1LI2, SYNJ1, MAPK1, and ARID4B are highly expressed in normal tissues, and CLOCK, SETD2, ZNF654, XIAP, MIS18BP1, and MBTPS2 are highly expressed in prostate cancer tissues (**Figure 9B**). These results were consistent with our analysis.

## Discussion

The main source of energy for cells is glucose. Glucose is metabolized to pyruvate by glycolysis, and pyruvate is oxidatively metabolized to CO_2_ in the tricarboxylic acid (TCA) cycle, and a large amount of ATP is generated by oxidative phosphorylation. However, glycolysis is active in tumor cells, even when oxygen is abundant, and this is the Warburg effect. Many previous studies have proved aerobic glycolysis to prostate cancer progression, metastasis, and drug resistance (23–26). There is no previous literature on the analysis of aerobic glycolysis-related genes to predict the prognosis of prostate cancer. Therefore, in this study, we focused on finding aerobic glycolysis-related genes and exploring whether they could be used as prognostic markers.

ssGSEA analysis results demonstrate that aerobic glycolysis not only is associated with prostate cancer prognosis but also significantly correlates with multiple energy metabolism pathways. This may imply that aerobic glycolysis may influence how tumors metabolize energy. We used WGCNA to screen genes related to aerobic glycolysis, then screen genes by MM and GS, and network in which hub genes were more closely connected to other genes. This makes the screened genes as relevant as possible to aerobic glycolysis without losing important genes. In the end, we got a signature consisting of 14 genes for predicting prognosis. Whether in the training dataset or in independent datasets, AGRS has good performance in predicting prognosis, which indicates a clinical application.

To explore the underlying mechanisms of the AGRS model, GSEA was performed to explore KEGG pathways among the two risk groups. Aberrant activation of multiple immune-related pathways, including viral protein interaction with cytokine and cytokine receptor, natural killer cell-mediated cytotoxicity, IL-17 signaling pathway, Th1- and Th2-cell differentiation, Th17-cell differentiation, T-cell receptor signaling pathway, leukocyte transendothelial migration, and PD-L1 expression and PD-1 checkpoint pathway in cancer, in high-risk groups is associated with worse prognosis in prostate cancer, which is consistent with immune cell analysis among different risk groups. High-risk groups had more abundant immune cell infiltration. However, T cells regulatory (Tregs) are also significantly higher than those in the low-risk group, so although the immune cells in the high-risk group are more abundant, most of them have lost their normal functions, or have become accomplices of tumors.

In our genomic mutation analysis, we found a higher rate of gene mutation in the high-risk group and identified specific gene mutations that were associated with aerobic glycolysis. NLRP3 has been shown by many studies to be related to the activation of inflammasomes in macrophages (27–29). JAK1 was found to have a higher mutation rate in the high-risk group, which is consistent with the GSEA results showing that the JAK stat signaling pathway was enriched in the high-risk group.

In addition, we analyzed the differences in metabolic genes between different subgroups, and analyzed the enrichment pathways of differentially metabolized genes, and found that the high-risk group was enriched in two drug metabolism-related pathways, which also indicated that maybe AGRS could be used to predict chemotherapy drug sensitivity. Based on predictions from GCP data, we found that the high-risk patients were more sensitive to the anticancer drugs etoposide, cisplatin, and rapamycin, and the low-risk patients were more sensitive to the anticancer drugs bicalutamide and 5-fluorouracil. Furthermore, the CMap database was employed to identify small-molecule drugs for PC. The drugs include PKC activator (ingenol and prostratin), ATPase inhibitor (cinobufagin), DNA methyltransferase inhibitor (azacitidine), DNA synthesis inhibitor (anisomycin), IKK inhibitor (BX-795), HSP inducer (homosalate), and A exonuclease inhibitor (mirin). These potential therapeutic agents may kill cancer cells through the aerobic glycolysis approach.

In the above analysis, we found that a variety of immune cells differed between the two risk subgroups. We therefore assessed whether AGRS could predict immunotherapy response. Interestingly, we found that the 23 immune checkpoint molecules we found were all highly expressed in the high-risk group. Furthermore, patients in the high-risk subgroup presented with higher IPS scores. The immunogenicity of two risk subgroups was evaluated by IPS analysis. Higher IPS scores are positively correlated to the increased immunogenicity. The above results indicate that patients in the high-risk group have a better response to immunotherapy.

Our research also inevitably has some limitations. Our analysis data are for tumor tissue as a whole, but tumor tissue contains not only cancer cells but also other non-cancer cells such as immune cells.

## Conclusion

Our study illustrates the crucial role of aerobic glycolysis in PC. An aerobic glycolysis gene-related prognostic model has been established and has good performance. AGRS can also be used to guide a patient’s drug treatment strategy.

## Data availability statement

The datasets presented in this study can be found in online repositories. The names of the repository/repositories and accession number(s) can be found in the article/**Supplementary Material**.

## Ethics statement

This study was reviewed and approved by The Ethics Committee of Zhejiang Provincial People’s Hospital, People’s Hospital of Hangzhou Medical College. Written informed consent to participate in this study was provided by the participants’ legal guardian/next of kin.

## Author contributions

WH and EL designed experiments. The preliminary preparation work and PCR were carried out by WH. EL contributed to the statistical analysis using R and edited the manuscript. XH helped perform the analysis with constructive discussions. All authors contributed to the article and approved the submitted version.

## Funding

This work was supported by the Zhejiang Provincial Natural Science Foundation of China under Grant No. LQ18H160022 and the Medical and Health Science and Technology Project of Zhejiang Province under Grant No. 2019KY272.

## Acknowledgments

The authors thank the Zhejiang Provincial Natural Science Foundation of China under Grant No. LQ18H160022 and the Medical and Health Science and Technology Project of Zhejiang Province under Grant No. 2019KY272.

## Conflict of interest

The authors declare that the research was conducted in the absence of any commercial or financial relationships that could be construed as a potential conflict of interest.

## Publisher’s note

All claims expressed in this article are solely those of the authors and do not necessarily represent those of their affiliated organizations, or those of the publisher, the editors and the reviewers. Any product that may be evaluated in this article, or claim that may be made by its manufacturer, is not guaranteed or endorsed by the publisher.

## Abbreviations

PC, Prostate cancer; BCR, biochemical recurrence; PSA, prostate-specific antigen; AG, aerobic glycolysis; TCGA, The Cancer Genome Atlas; WGCNA, weighted genes co-expression network analysis; GEO, Gene-Expression Omnibus; OS, over survival; TPM, transcripts per million; TCIA, The Cancer Immunome Atlas; AGRS, aerobic glycolysis risk score; ssGSEA, single-sample gene set enrichment analysis; KM, Kaplan-Meier; the ROC, received operating characteristic; AUC, the area under the receiver operating characteristic curve; KEGG, Kyoto Encyclopedia of Genes and Genomes; IC50, the half maximal inhibitory concentration; CMap, the Connectivity Map; HPA, Human Protein Atlas; MM, Module membership; GS, Gene significance.
