# Supplementary figures and images for: Comprehensive analysis of aerobic glycolysis-related genes for prognosis, immune features and drug treatment strategy in prostate cancer
Source: Front Oncol. 2022 Sep 29;12:905888. doi: 10.3389/fonc.2022.905888 (PMC9556868; doi:10.3389/fonc.2022.905888)

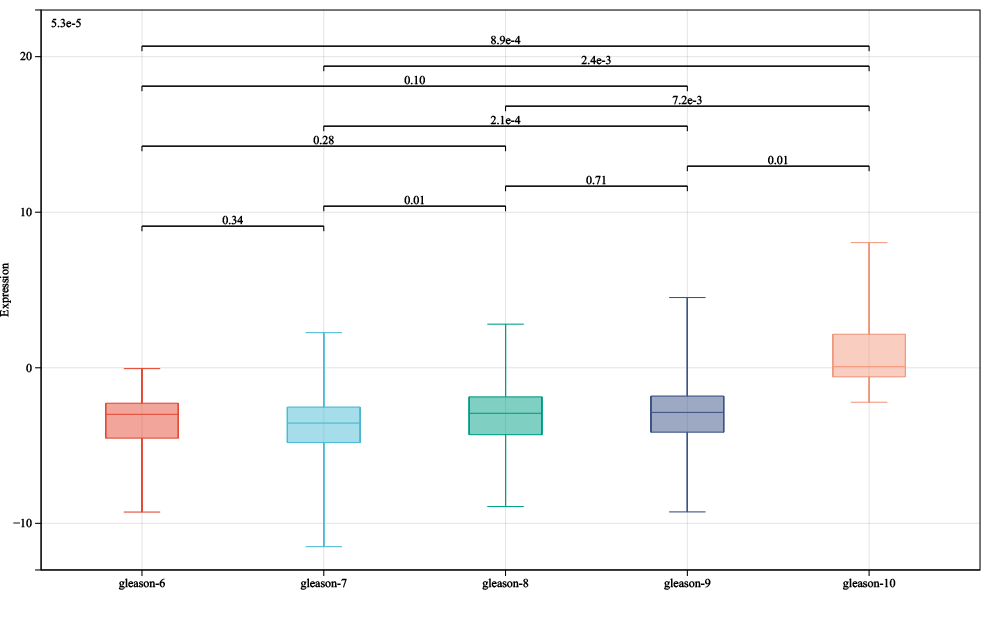

Supplement: Supplementary file 1 [file Image_1.tif]

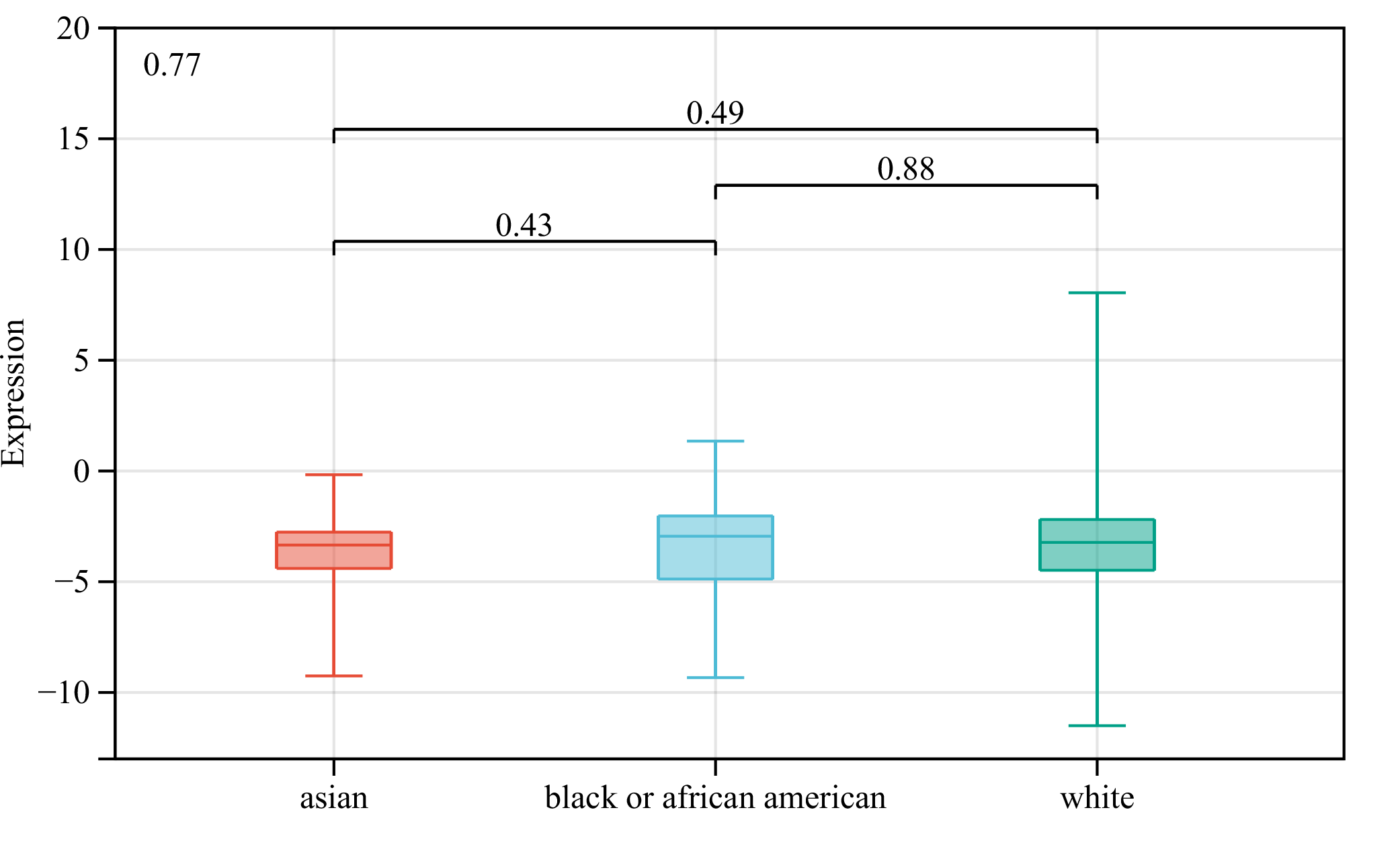

Supplement: Supplementary file 2 [file Image_2.tif]

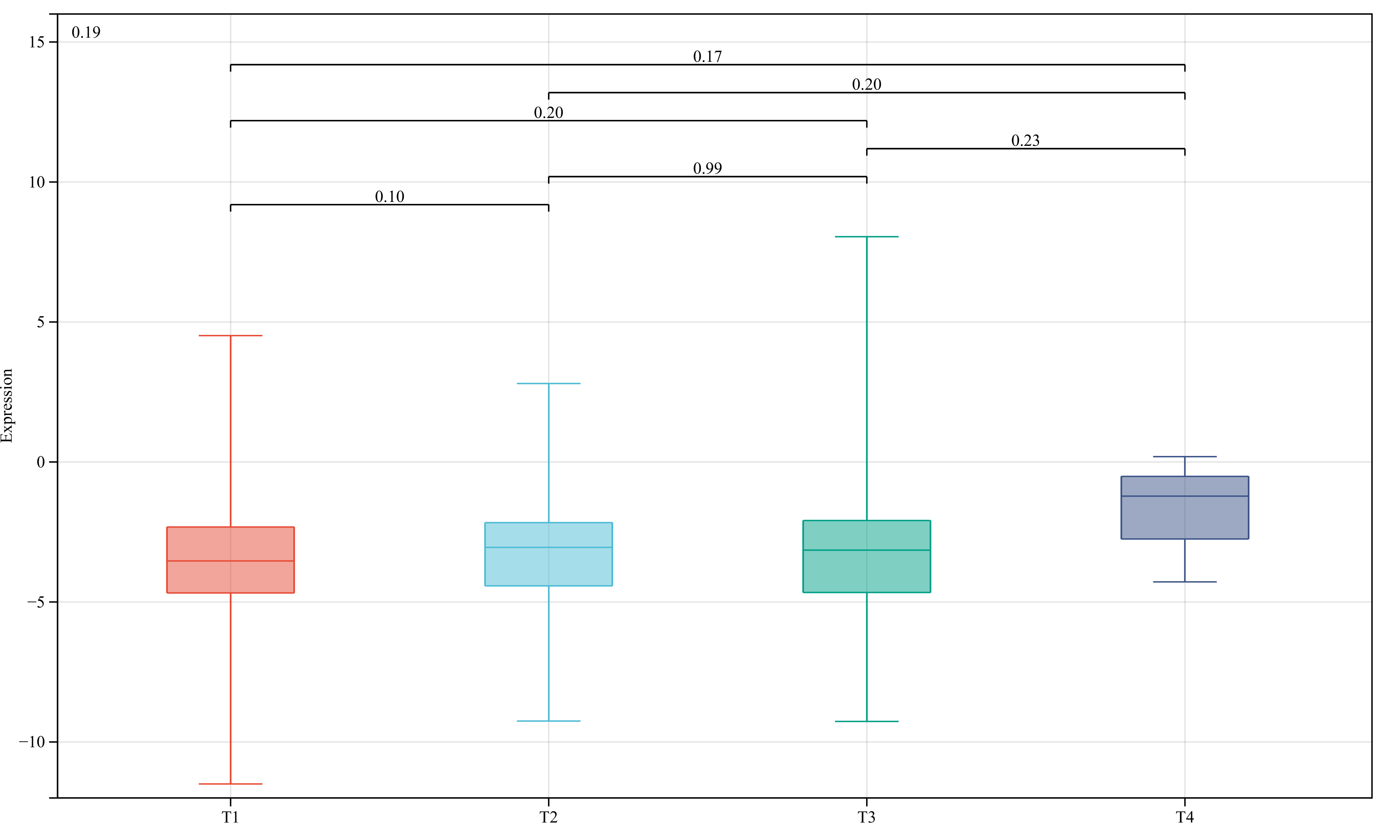

Supplement: Supplementary file 3 [file Image_3.tif]

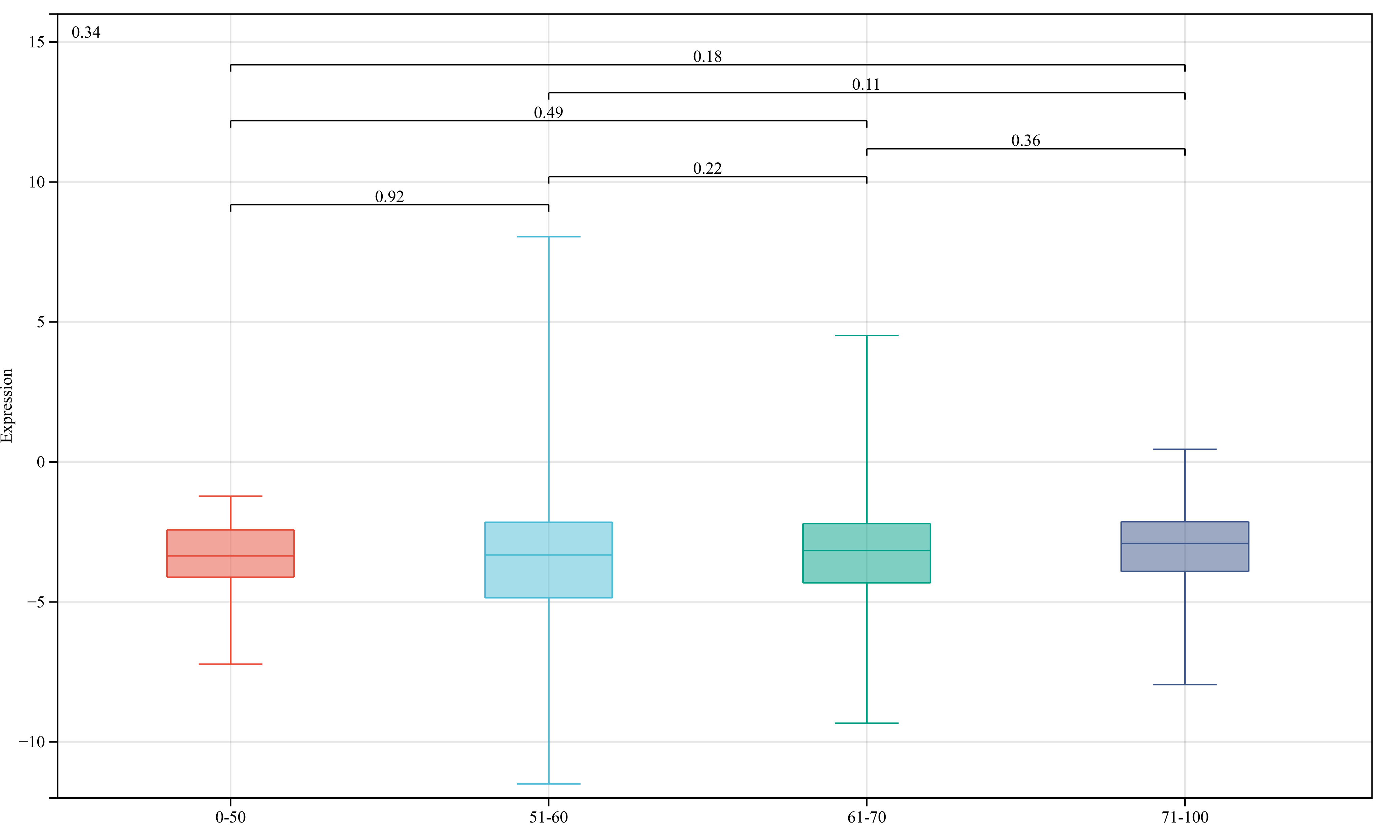

Supplement: Supplementary file 4 [file Image_4.tif]
